# Acrolein contributes to human colorectal tumorigenesis through the activation of RAS-MAPK pathway

**DOI:** 10.1038/s41598-021-92035-z

**Published:** 2021-06-15

**Authors:** Hong-Chieh Tsai, Han-Hsing Tsou, Chun-Chi Lin, Shao-Chen Chen, Hsiao-Wei Cheng, Tsung-Yun Liu, Wei-Shone Chen, Jeng-Kai Jiang, Shung-Haur Yang, Shih-Ching Chang, Hao-Wei Teng, Hsiang-Tsui Wang

**Affiliations:** 1 Department of Neurosurgery, Linkou Chang Gung Memorial Hospital, Taoyuan, 333 Taiwan; 2 School of Traditional Chinese Medicine, Chang Gung University, Taoyuan, 333 Taiwan; 3 Institute of Food Safety and Health Risk Assessment, National Yang-Ming University, Taipei, Taiwan; 4 Institute of Food Safety and Health Risk Assessment, National Yang Ming Chiao Tung University, Taipei, Taiwan; 5 Kim Forest Enterprise Co., Ltd., Taipei, Taiwan; 6 School of Medicine, National Yang-Ming University, Taipei, Taiwan; 7 School of Medicine, National Yang Ming Chiao Tung University, Taipei, Taiwan; 8 Division of Colon and Rectum Surgery, Department of Surgery, Taipei Veterans General Hospital, Taipei, Taiwan; 9 Institute of Pharmacology, College of Medicine, National Yang-Ming University, Taipei, Taiwan; 10 Institute of Pharmacology, College of Medicine, National Yang Ming Chiao Tung University, Taipei, Taiwan; 11 Department of Surgery, National Yang Ming Chiao Tung University Hospital, Yilan, Taiwan; 12 Division of Medical Oncology, Department of Oncology, Taipei Veterans General Hospital, Taipei, Taiwan

**Keywords:** Cancer, Environmental sciences, Oncology

## Abstract

Colorectal cancer (CRC) is one of the most well-known malignancies with high prevalence and poor 5-year survival. Previous studies have demonstrated that a high-fat diet (HFD) is capable of increasing the odds of developing CRC. Acrolein, an IARC group 2A carcinogen, can be formed from carbohydrates, vegetable oils, animal fats, and amino acids through the Maillard reaction during the preparation of foods. Consequently, humans are at risk of acrolein exposure through the consumption of foods rich in fat. However, whether acrolein contributes to HFD-induced CRC has not been determined. In this study, we found that acrolein induced oncogenic transformation, including faster cell cycling, proliferation, soft agar formation, sphere formation and cell migration, in NIH/3T3 cells. Using xenograft tumorigenicity assays, the acrolein-transformed NIH/3T3 clone formed tumors. In addition, cDNA microarray and bioinformatics studies by Ingenuity Pathway Analysis pointed to the fact that RAS/MAPK pathway was activated in acrolein-transformed clones that contributed to colon tumorigenesis. Furthermore, acrolein-induced DNA damages (Acr-dG adducts) were higher in CRC tumor tissues than in normal epithelial cells in CRC patients. Notably, CRC patients with higher levels of Acr-dG adducts appeared to have better prognosis. The results of this study demonstrate for the first time that acrolein is important in oncogenic transformation through activation of the RAS/MAPK signaling pathway, contributing to colon tumorigenesis.

## Introduction

Colorectal cancer (CRC) is the third most frequent neoplasm worldwide (https://www.wcrf.org). Although diagnosis and therapy have advanced significantly over the past 10 years, its prevalence is rising, and the 5-year survival rate is poor^1^. CRC has become a significant issue for healthcare in Asian countries, with a two to fourfold increase in incidence over the rate decades ago. CRC emerges from benign neoplasms and develops into adenocarcinomas through a stepwise histological progression sequence, continuing from either adenomas or hyperplastic polyps/serrated adenomas. Genetic modifications have been related to specific steps in this adenoma-carcinoma sequence and are believed to drive the histological progression of CRC^2,3^. CRC arises from an association of genetic and environmental factors, and it has been identified as involving multiple cell signaling pathways, such as Wnt, epidermal growth factor receptor/mitogen-activated protein kinase (EGFR/MAPK), tumor protein 53 (TP53), phosphoinositide 3-kinase (PI3K), and transforming growth factor beta (TGFβ)/SMAD^4,5^. Epidemiologic studies have shown that lifestyle and dietary habits impact the danger of developing CRC^6^. In particular, the intake of foods rich in fat and with low fiber content (known as a high-fat diet (HFD) or Western-style diet) increases the odds of developing CRC^7–9^.

Acrolein (2-propenal), the most reactive α, β-unsaturated aldehyde, is a highly mutagenic and highly oxidizing environmental toxin^10^. The most well-studied source of acrolein exposure is tobacco smoking, which has been shown to be associated with oral, lung and bladder cancer^11–16^. IARC working groups re-evaluated acrolein as probably carcinogenic to humans (Group 2A) on the basis of sufficient evidence of carcinogenicity in experimental animals and strong mechanistic evidence^17^. Nonetheless, its dietary exposure and consequences are under investigation. Acrolein can be formed from carbohydrates, vegetable oils, animal fats, and amino acids during the preparation of foods^18^. It is formed during the Maillard reaction as a result of the conversion of amino acids^19,20^ and the oxidative deamination of polyamines^21^. Likewise, acrolein can be detected in the emissions of varieties of heated or overheated cooking oils and as such is found abundantly in fried food such as French fries^22^. Additionally, recent reports demonstrated that gut microbial glycerol metabolism prompts the formation of reuterin, which is an additional source of endogenous acrolein^23^. Reuterin is an antimicrobial multicomponent system comprising 3-hydroxypropionaldehyde, its dimer and hydrate, and acrolein. Our recent studies have shown that exposure to acrolein following consumption of fried food influences local oral cavity homeostasis^24^. Consequently, humans are in danger of acrolein exposure through consumption of food rich in high fat^25,26^.

Although the association between HFD and CRC risk has been known for quite a while^27–29^, the exact mechanisms underlying HFD-induced colon cancer risk and recurrence have remained unclear. The large-scale connections of dietary components with one another and with metabolism make it hard to specifically recognize the components in HFD that might cause CRC^8^. Since acrolein can be produced during the preparation of foods^18^, we aimed to investigate the role of acrolein in CRC tumorigenesis. In the present study, we determined the effect of acrolein on oncogenic transformation using NIH/3T3 cells with xenograft tumorigenesis mouse models. Furthermore, cDNA microarray analysis with Ingenuity Pathway Analysis (IPA) was performed in acrolein-transformed NIH/3T3 cells. Finally, acrolein-induced DNA damages (Acr-dG adducts) were analyzed in tumor tissues and normal epithelial tissues of CRC patients, and the levels of Acr-dG adducts were associated with tumor characteristics and CRC patient survival.

## Results

### Acrolein treatment induced cell proliferation, anchorage-independent activity, spheroid formation ability and cell migration capacity

**Figure 1:**
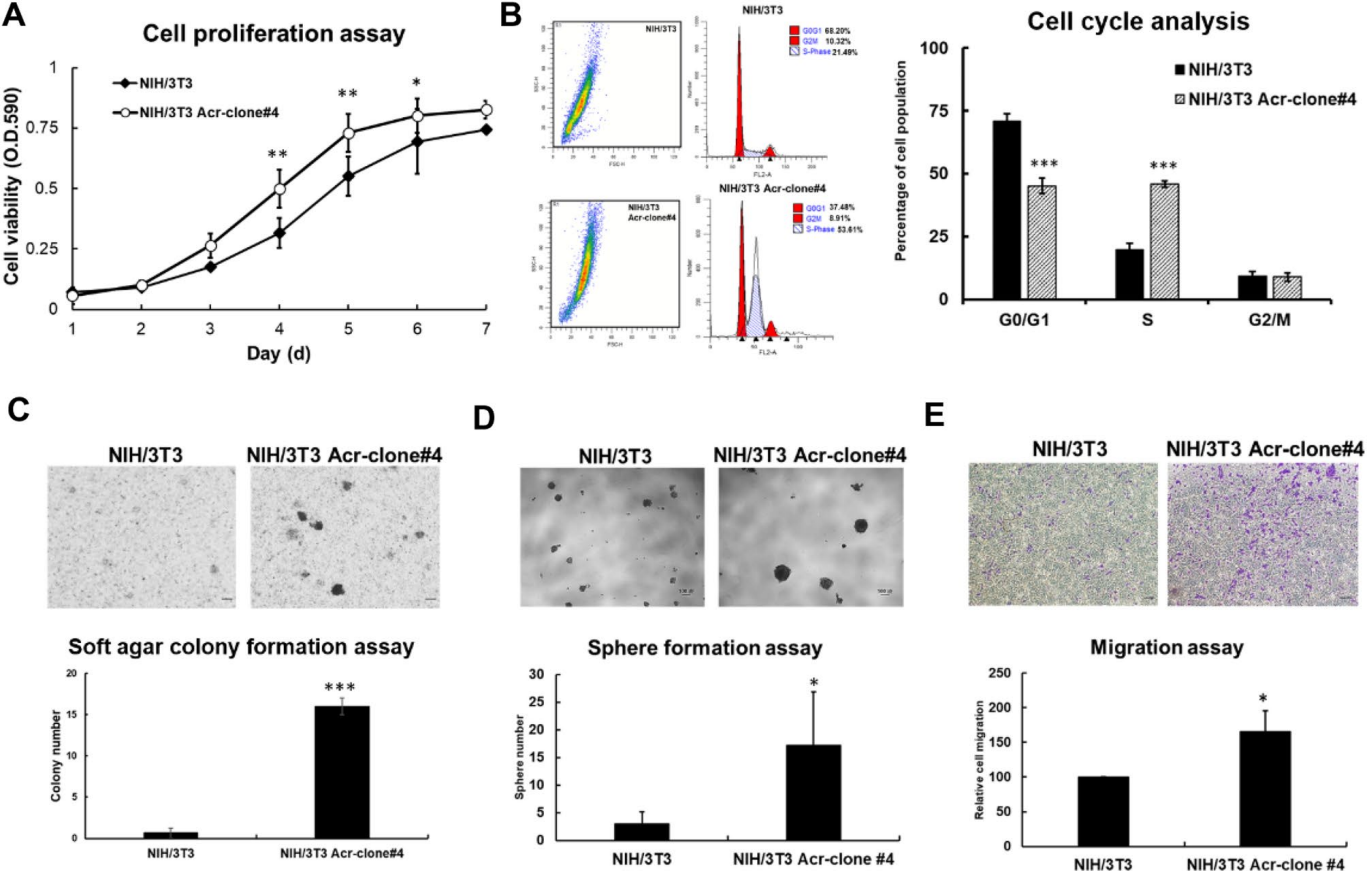
Acrolein induced oncogenic transformation using a cellular model of NIH/3T3 cells. NIH/3T3 cells were treated with acrolein (Acr, 7.5 μM) for one month and named NIH/3T3 Acr-clone#. **(A)** Cell proliferation of NIH/3T3 Acr-clone #4 cells was analyzed using MTT assays. **(B)** Cell cycle progression of NIH/3T3 Acr-clone #4 cells was analyzed using cell cycle analysis with PI staining. **(C)** Soft agar anchorage-dependent cell growth of NIH/3T3 Acr-clone #4 was analyzed using a soft agar assay. **(D)** The spheroid formation ability of NIH/3T3 Acr-clone #4 cells was analyzed on ultralow attachment plates. **(E)** The cell migration activity of NIH/3T3 Acr-clone #4 cells was analyzed using Transwell migration analysis. Scale bar: 100 µm. NIH/3T3 Acr-clone #4 had the highest cell transformation activity. Student’s *t* tests were used to determine statistical significance, and two-tailed p-values are shown. *p < 0.05, **p < 0.01, ***p < 0.005 compared with NIH/3T3 parental cells.

To determine the potential role of acrolein in oncogenic transformation, we treated NIH/3T3 cells with a low dose of acrolein (7.5 μM, IC_10_) for one month and selected NIH/3T3 Acr clones #1–#7 (Supplementary Fig. 1A). The soft agar colony formation activity of these seven clones was analyzed, and the results showed that NIH/3T3 Acr-clones #3, #4 and #6 formed more colonies than the others (Supplementary Fig. 1B). Cell proliferation analysis showed that NIH/3T3 Acr-clone #4 (doubling time = 31.0 h) had faster proliferation than parental cells (doubling time = 39.4 h); however, NIH/3T3 Acr-clone #3 (doubling time = 55.0 h) or NIH/3T3 Acr-clone#6 (doubling time = 42.9 h) showed the opposite phenomenon (Fig. 1A, Supplementary Fig. 1C). Therefore, we selected NIH/3T3Acr-clone #4 for subsequent analysis. Consistently, cell cycle analysis showed that the ratio of NIH/3T3 Acr-clone#4 cells in S phase was markedly higher than that in parental cells (Fig. 1B, Supplementary Fig. 1D), indicating that acrolein promotes S-phase DNA synthesis and accelerates cell proliferation. Anchorage-independent activity (Fig. 1C) in NIH/3T3 Acr-clone#4 cells was also increased compared to parental NIH/3T3 cells using a soft agar colony formation assay. Spheroid formation ability on ultralow attachment plates of NIH/3T3 Acr-clone#4 was also enhanced (Fig. 1D). In addition, NIH/3T3 Acr-clone#4 cells showed enhanced migration capacity compared with NIH/3T3-mock cells (Fig. 1E) using a Transwell assay. However, the drug sensitivity of NIH/3T3 Acr-clone#4 toward chemotherapeutic agents such as oxaliplatin and 5-FU was similar to that of parental NIH/3T3 cells (Supplementary Fig. 2). These results suggest that acrolein increases the cell cycle rate, proliferation, colony formation activity, spheroid formation ability and cell migration capacity.

### NIH/3T3 Acr-clone#4 formed tumors in xenograft nude mice

**Figure 2:**
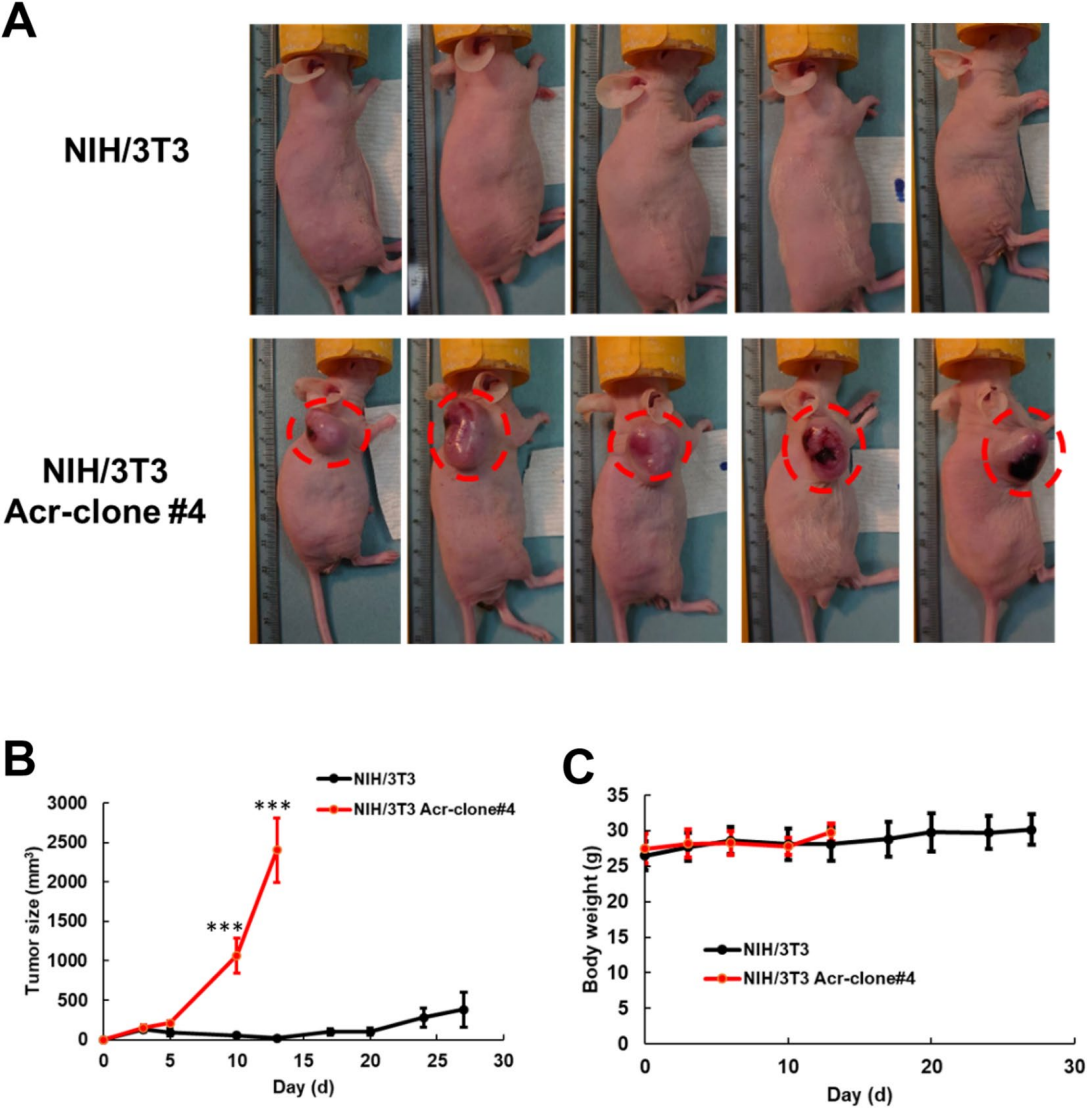
Xenograft mouse model of acrolein-transformed clones. **(A)** Overall view of tumors formed by acrolein-transformed NIH/3T3 cell clone (Acr-clone#4). Tumors in nude mice were observed after injection with acrolein-transformed clone #4, whereas none were observed after injection with mouse fibroblasts or NIH/3T3 cells. **(B)** Tumor growth curves and **(C)** body weight of nude mice in different experimental groups (n = 5). Student’s *t* tests were used to determine statistical significance, and two-tailed p-values are shown. ***p < 0.005 compared with NIH/3T3 parental cells.

Our in vitro results indicate that acrolein can transform normal mouse NIH/3T3 fibroblasts into malignant cells. To confirm its tumorigenic potential, we performed in vivo studies of tumor xenografts in nude mice using parental NIH/3T3 cells as the negative control. NIH/3T3 Acr-clone#4 and parental NIH/3T3 cells were injected subcutaneously into the right axillary fossa (5 × 10^6^ cells/animal). Three weeks after injecting NIH/3T3 Acr-clone#4 into nude mice, nodular neoplasms could be observed, while tumors were obvious at 10 days, whereas the parental NIH/3T3 cells failed to form any tumors (Fig. 2A). Tumors formed by NIH/3T3 Acr-clone#4 cells were observed, and their volumes and growth curves were calculated for 4 weeks after the tumors could be observed (Fig. 2B,C). These data further indicate that acrolein leads to oncogenic transformation in vivo.

### Acrolein induced the RAS/MAPK signaling pathway in CRC tumorigenesis using ingenuity pathway analysis (IPA)

**Figure 3:**
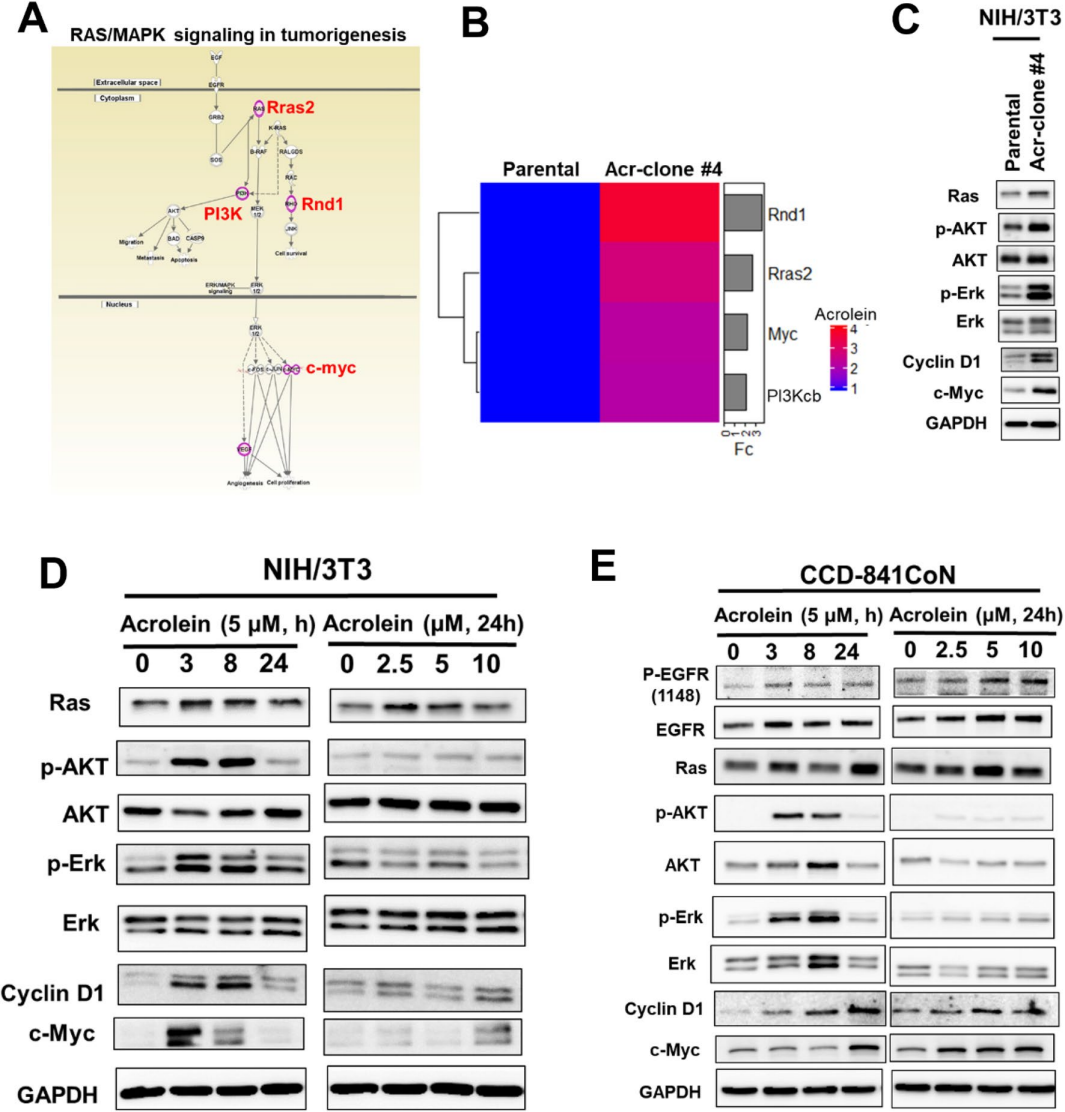
Ingenuity Pathway Analysis (IPA) of gene expression profiles in acrolein-transformed clones. **(A)** Bioinformatic analysis of the differentially expressed genes (DEGs, fold change ≥ 2) was performed using IPA, and canonical pathway analysis for gene expression profiles in acrolein-transformed clone #4 (Acr-clone #4) is shown. **(B)** Heatmap of 4 genes (Rnd1, Rras2, Myc and PI3Kcb) in acrolein-transformed clone #4 compared to parental NIH/3T3 cells. FC, fold-change, indicates RNA expression fold change between NIH/3T3 Acr-clone#4 and parental cells. **(C)** Western blot analysis of the RAS/ERK and AKT pathways in acrolein-transformed clone #4 compared to parental NIH/3T3 cells. **(D,E)** Dose and time effects of acrolein on RAS expression, AKT activation, ERK activation, cyclin D1 and c-myc expression in NIH/3T3 cells **(D)** and CCD-841CoN cells **(E)** were analyzed using Western blot analysis. For dose and time effects, cells were treated with different concentrations of acrolein (0–10 μM) for 24 h or acrolein (5 μM) for 3–24 h, respectively. Original Western blots of **(C–E)** are shown in Supplementary Fig. 3A–C.

**Figure 4:**
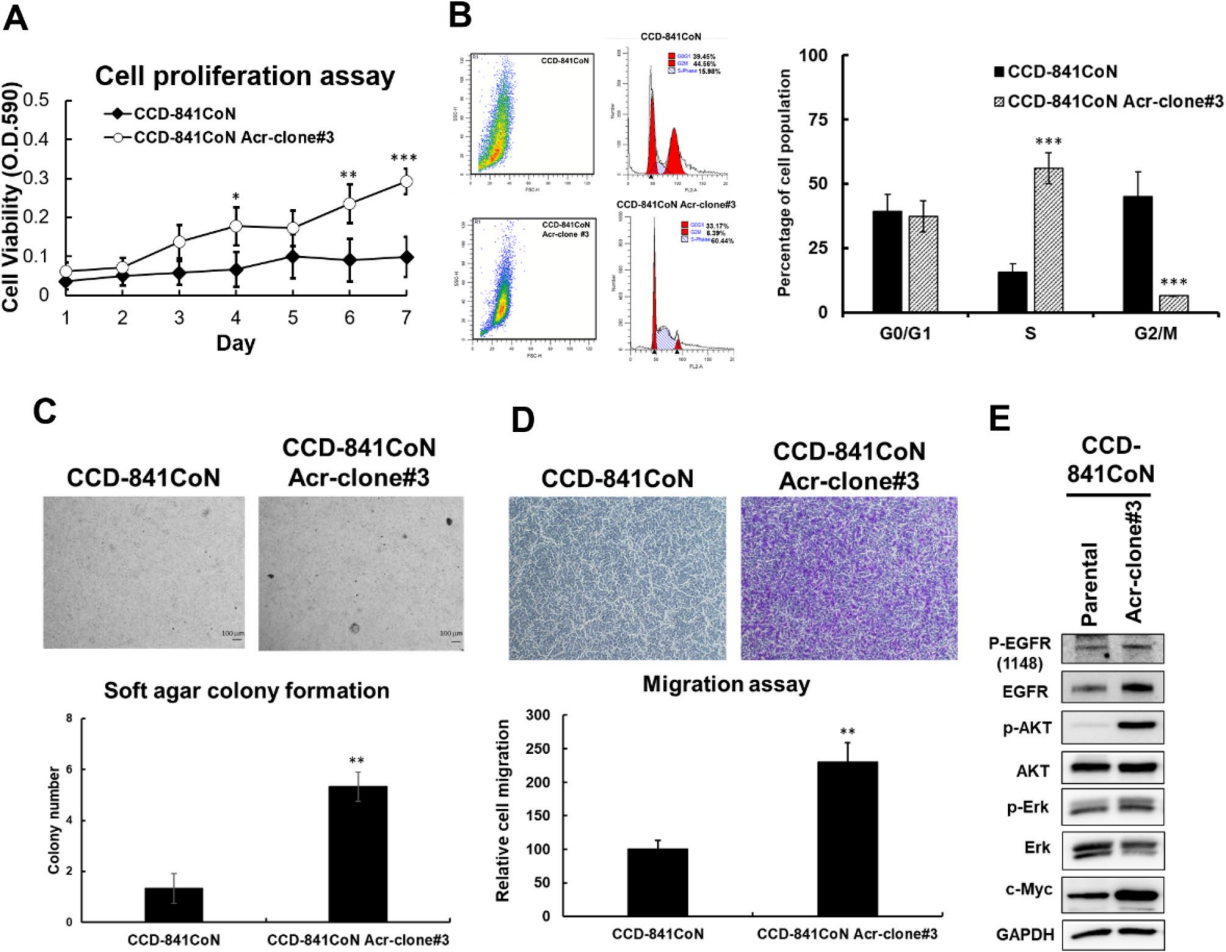
Acrolein induced oncogenic transformation in CCD-841CoN cells. CCD-841CoN cells were treated with acrolein (Acr, 7.5 μM) for one month and named CCD-841CoN Acr-clone#3. **(A)** Cell proliferation of CCD-841CoN Acr-clone#3 was analyzed using MTT assays. **(B)** Cell cycle progression of CCD-841CoN Acr-clone#3 was analyzed using cell cycle analysis with PI staining. **(C)** Soft agar anchorage-dependent cell growth of CCD-841CoN Acr-clone#3 was analyzed using a soft agar assay. Scale bar: 100 µm. **(D)** The cell migration activity of CCD-841CoN Acr-clone#3 cells was analyzed using Transwell migration analysis. Student’s t tests were used to determine statistical significance, and two-tailed p-values are shown. *p < 0.05, **p < 0.01, ***p < 0.005 compared with CCD-841CoN parental cells. **(E)** Western blot analysis of the RAS/ERK and AKT pathways in acrolein-transformed clones compared to parental CCD-841CoN cells. Original Western blots of **(E)** are shown in Supplementary Fig. 3D.

To determine the underlying mechanisms by which acrolein induced oncogenic transformation, cDNA microarray analysis with IPA was performed in NIH/3T3 Acr-clone#4 cells (Fig. 3A). The results showed that four genes (Rnd1, Rras2, myc and PI3Kcb) involved in the RAS/MAPK signaling pathway were upregulated in acrolein-transformed clone #4 (NIH/3T3 Acr-clone #4) (Fig. 3B, Supplementary Table 1). These results were confirmed using Western blot analysis (Fig. 3C, Supplementary Fig. 3A). Furthermore, we also found that acrolein activated the RAS/MAPK signaling pathway and increased c-myc in NIH/3T3 cells and the human normal colon epithelium CCD-841CoN (Fig. 3D,E, Supplementary Fig. 3B,C). Intriguingly, acrolein induced cell proliferation, colony formation activity and cell migration capacity in CCD-841CoN cells (Fig. 4A,D). Activation of the RAS/MAPK signaling pathway was also observed in the CCD-841CoN Acr clone (Fig. 4E, Supplementary Fig. 3D) as well as human colon cancer cell lines, SW480 and HCT116 (Supplementary Fig. 4). These results indicate that acrolein induced oncogenic transformation through activation of the RAS/MAPK pathways.

### Immunohistochemistry analysis of acrolein-DNA (Acr-dG) levels in human colon cancers

**Figure 5:**
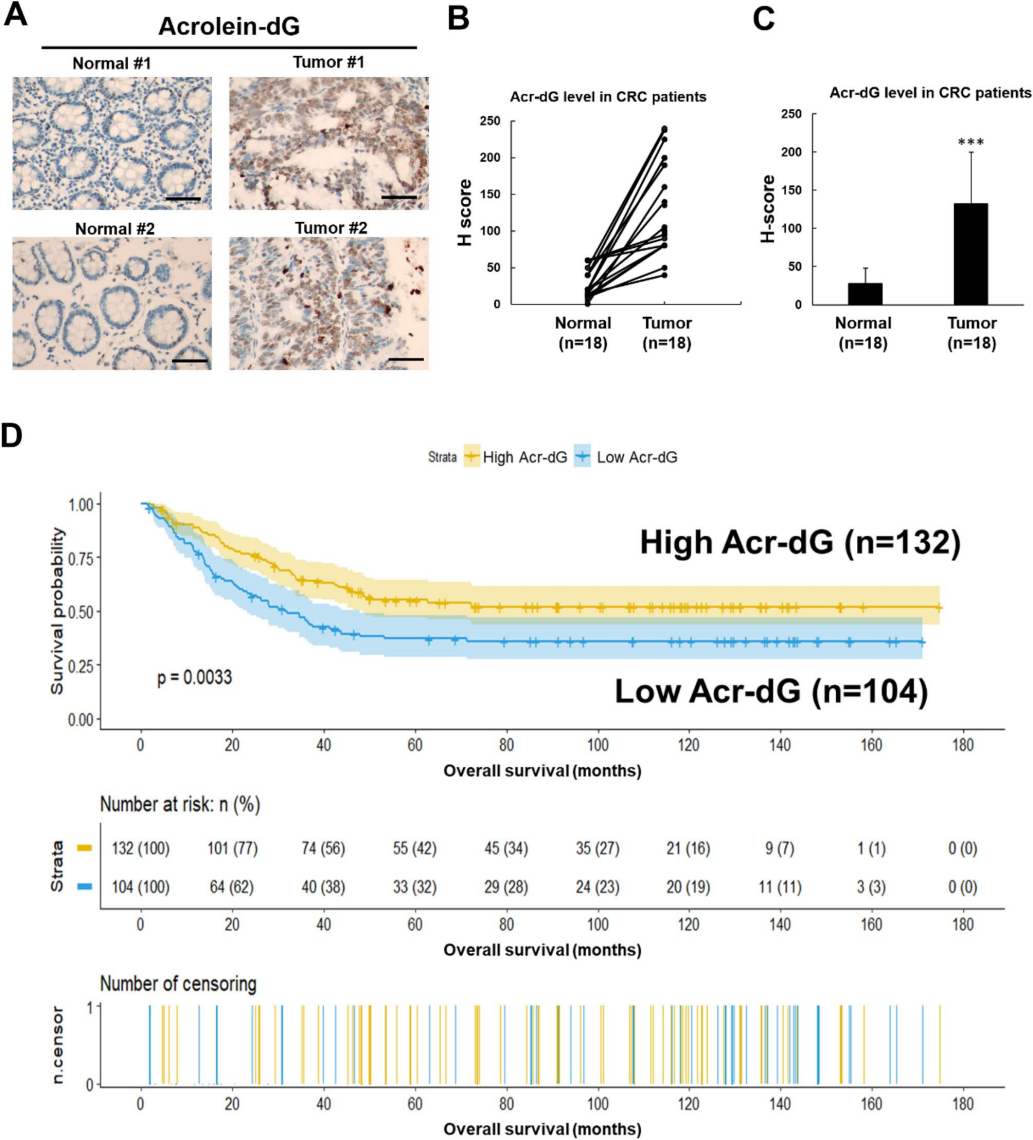
Immunohistochemical staining for Acr-dG adducts in eighteen CRC patients and Kaplan–Meier survival analysis of the high and low Acr-dG expression groups in CRC patients. **(A)** Representative image of Acr-dG adducts in normal epithelial cells adjacent to CRC tumor tissues (magnification, × 400). Scale bar: 50 μm. **(B,C)** Quantification of Acr-dG adducts in normal epithelial cells adjacent to CRC tumor tissues. **(B)** Relative Acr-dG levels between normal and tumor tissues in 18 individual CRC patients. **(C)** Average Acr-dG levels in normal tissues and tissues from 18 CRC patients. Student’s *t* tests were used to determine statistical significance, and two-tailed p-values are shown. *p < 0.05, **p < 0.01, ***p < 0.005 compared between tumor tissues and normal tissues. **(D)** IHC analysis of Acr-dG levels in CRC tissues (n = 236) was performed as described in the “Materials and Methods”. The Kaplan–Meier survival analysis survival plot was analyzed with the R software (R version 4.1.0). The number at risk and the number of censors in study cases are listed. Additionally, the endpoint was cancer-specific death.

Acrolein can react with DNA-inducing modifications, which, if not repaired, can result in mutations and lead to cancer development. Acrolein has been shown to produce propano-2’-deoxyguanosine (Acr-dG) adducts in human cells^15,30,31^. Acr-dG adducts are mutagenic and induce mainly G to T and G to A mutations^15,31–40^. To further investigate whether acrolein contributes to colon cancer formation, we analyzed Acr-dG adduct expression in CRC tissues and normal epithelial cells adjacent to tumor tissues using immunohistochemical (IHC) staining. The results showed that Acr-dG adduct levels were mainly located in the nucleus and were higher in CRC tumor tissue than in normal epithelial cells in 18 CRC patients (Fig. 5A–C). Based on our cDNA microarray data, c-myc was upregulated in acrolein-transformed cell clones (Fig. 3C). We further analyzed c-myc levels in CRC tissues and normal epithelial cells in the same patients using IHC staining (Supplementary Fig. 5). Similar to Acr-dG adduct levels, higher c-myc levels were observed in CRC tumor tissues than in normal epithelial cells.

### Higher Acr-dG expression is associated with improved survival in CRC patients

We further evaluated the effect of Acr-dG expression on CRC characteristics and patient survival. The demographic data are shown in Table 1. Of 236 CRC patients, the majority had adenocarcinoma (211/236, 89.4%), and advanced stage III and IV cancer was diagnosed in 64% (151/236) of patients. The expression of Acr-dG was defined as low (H score < 50) or high (H score ≥ 50) based on the results showing Acr-dG levels in CRC tumor tissues and normal epithelia (Fig. 5B,C). Kaplan–Meier survival analysis revealed that the median survival was 103.4 months for CRC patients with high Acr-dG levels and 74.3 months for CRC patients with low Acr-dG levels, indicating that high expression of Acr-dG in tumor tissue was associated with better CRC patient overall survival (p = 0.0033, Fig. 5D). In addition, high expression of Acr-dG was inversely correlated with clinical stages and grades using chi-square analysis (Table 1). The impact of confounders on Acr-dG was analyzed using Cox proportional hazards models, as shown in Supplementary Tables 2–3. Before the multiple Cox regression analysis (Supplementary Table 2), the proportional hazards assumption test for a Cox regression model was performed (Supplementary Table 3). The Schoenfeld residuals are shown for our multivariable Cox regression model fit to the dataset with confounders (age, location, sex, acrolein, stage, grade and LVSI), as shown in Supplementary Table 4. Based on these results, Acr-dG was not an independent prognostic factor after controlling for age, location, sex, acrolein, stage, grade and LVSI. Taken together, our data suggest that CRC patients with higher Acr-dG expression in tumor tissues have a better prognosis.

**Table 1:** Clinical characteristics of CRC patients.

		Acr-dG levels				P value
		Low (h score < 50)		High (h score > = 50)		
		Count	%	Count	%	
Age	Mean ± SD	64.7 ± 13.8		63.8 ± 12.6		
	< 70 years	56	53.8%	83	62.9%	0.161
	≥ 70 years	48	46.2%	49	37.1%	
Sex	Female	40	38.5%	44	33.3%	0.414
	Male	64	61.5%	88	66.7%	
Location	Left	43	41.3%	60	45.5%	0.528
	Right	61	58.7%	72	54.5%	
Pathology	Adenocarcinoma	91	87.5%	120	90.9%	< 0.001^a^
	Carcinoma	1	1.0%	0	.0%	
	Mucinous adenocarcinoma	12	11.5%	12	9.1%	
Clinical Stage (AJCC 6th)	I	6	5.8%	12	9.1%	0.041*
	II	26	25.0%	41	31.1%	
	III	26	25.0%	44	33.3%	
	IV	46	44.2%	35	26.5%	
Grade	Low	90	86.5%	126	95.5%	0.015*
	High	14	13.5%	6	4.5%	
Mucinous component	No	64	61.5%	83	62.9%	0.833
	Yes	40	38.5%	49	37.1%	
LVSI	No	74	71.2%	107	81.1%	0.074
	Yes	30	28.8%	25	18.9%	
PNI	No	37	88.1%	38	97.4%	0.203^a^
	Yes	5	11.9%	1	2.6%	
Death event	No	40	38.5%	73	55.3%	0.010*
	Yes	64	61.5%	59	44.7%	

*AJCC* American Joint Committee on Cancer, *LVSI* lymph-vascular space invasion, *PNI* perineural invasion.

*P < 0.05, the chi-square statistic is significant at the 0.05 level.

^a^Fisher's exact test.

## Discussion

Acrolein is the most reactive α, β-unsaturated aldehyde present in tobacco smoke, in ambient air pollution, and in some cooking oils heated to a high temperature^18^. Acrolein was previously evaluated as a group 3 carcinogen by the IARC Working Group in 1995; however, it was re-evaluated as probably carcinogenic to humans (Group 2A)^17^. Our previous studies have also supported that acrolein is associated with oral, lung and bladder cancer^12,13,15,16^. Furthermore, our current studies have shown that individuals could be exposed to acrolein from consuming fried food^24^. Although the association between HFD and CRC risk has been known for quite a while^27–29^, the exact mechanisms underlying HFD-induced colon cancer risk and recurrence remain unclear. In the present study, our results showed that acrolein induced oncogenic transformation in NIH/3T3 cells in vitro and in vivo. The underlying mechanism was through activation of the RAS/MAPK pathway, which contributes to colon carcinogenesis. Additionally, Acr-dG adducts were higher in CRC tumor tissues than in normal epithelial cells in CRC patients. These results suggest that acrolein may contribute to colon tumorigenesis. Furthermore, slot blot analysis showed increased Acr-dG levels in mouse colon tissues fed a HFD for 24 weeks compared to mice fed a normal diet (Supplementary Fig. 6). Furthermore, we found that acrolein-protein conjugates (Acr-PC) were increased in colon tissues of mice fed a HFD for 4–24 weeks (Supplementary Fig. 7), and activation of RAS/MAPK signaling was also observed in colon tissues of mice fed a HFD (data not shown). Acrolein contains a carbonyl group and an olefinic double bond and has been shown to react with cysteine, histidine, and lysine residues of proteins and with nucleophilic sites in DNA^41–43^. These results indicate that HFD induced acrolein production in the mouse colon and that acrolein may contribute to HFD-induced colon tumorigenesis.

In vivo exposure to acrolein in most situations is quite low, and the effects may differ from those seen at acutely toxic doses^10,42^. Acrolein, the most reactive α,β-unsaturated aldehyde, rapidly binds to and depletes cellular nucleophiles such as glutathione and reacts with proteins and DNA^41–43^. This reactivity is the basis for the cytotoxicity evident in all cells exposed to high concentrations of acrolein^44–47^. On the other hand, acrolein inhibits cell proliferation without causing cell death at low doses^48–50^. Previous studies have shown that the peak concentration of daily oral acrolein exposure has been estimated at 30 μg/kg bw^25^, which is approximately 7.5 μM based on a 70 kg adult with a 5 L blood volume. Therefore, we used a sublethal dose of acrolein (7.5 μM, IC10) to expose NIH/3T3 cells for one month to mimic in vivo conditions (Supplementary Fig. 1A). The results showed that acrolein was able to transform NIH/3T3 cells, and NIH/3T3 Acr-clones #3, #4 and #6 formed more colonies than the others (Supplementary Fig. 1B). However, cell proliferation analysis showed the opposite phenomenon (Fig. 1A, Supplementary Fig. 1C). Furthermore, NIH/3T3 Acr-clone#4 was able to form tumors, whereas no tumors were observed in mice inoculated with NIH/3T3 parental cells or NIH/3T3 Acr-clone#3 (data not shown) using a xenograft mouse model (Fig. 2). The mechanisms underlying acrolein-induced cell transformation may be related to the ability of acrolein to deplete cellular thiols or other nucleophiles and/or to effects on gene activation, either directly or subsequent to effects on redox-regulated transcription factors^10,42^. To explore the possible signaling involved in acrolein-induced oncogenic transformation, we used a cDNA microarray with IPA analysis, and the results showed that the RAS/MPAK pathway was the top of canonical pathway analysis (Fig. 3A).

Previous studies have shown that alterations in EGFR-related Ras-Raf-MAPK and PI3K-Akt pathways are involved in the pathogenesis of up to 55% and 15% of CRC, respectively^51^. Upregulation of c-myc protein plays an essential role in tumorigenesis through frequently altered kinase MAPK and RAS pathways in CRC^52^. In this study, we found that acrolein upregulated the RAS/MAPK pathway followed by overexpression of c-myc in both NIH/3T3 and colon cells, CCD-841coN (Figs. 3,4). Consistently, increased c-myc expression was also observed in these CRC tumor tissues (Supplementary Fig. 5), along with higher Acr-dG adducts in these tumor tissues (Fig. 5A–C). Acrolein is a highly reactive aldehyde reacting with dG of DNA to form Acr-dG adducts, which were shown to be mutagenic^15,31–40^. It is unclear whether acrolein induces mutations in RAS/MAPK pathways. These results showed that acrolein may be involved in colon tumorigenesis and that the underlying mechanism is possible through activation of the RAS/MAPK pathway and upregulation of c-myc.

Interestingly, we found that CRC patients with higher Acr-dG expression in tumor tissues had a better prognosis (Fig. 5D, Table 1). A possible explanation is that Acr-dG adducts are involved in the initiation of colon tumorigenesis; however, accumulating high amounts of Acr-dG adducts trigger cellular apoptosis. Acrolein can be produced through lipid peroxidation in fast dividing cells such as cancer cells^53^. Our previous studies have shown that hypoxia induces acrolein production, resulting in cellular apoptosis^54^. Furthermore, an inverse correlation between cell viability and relative Acr-dG adduct levels was observed in acrolein-treated NIH/3T3 cells (Supplementary Fig. 8). In addition, acrolein induced cytotoxicity in the colon cancer cell lines SW480 and HCT116 (Supplementary Fig. 9). This may explain why CRC patients with higher Acr-dG adduct levels were associated with better survival. However, the detailed mechanisms still need further investigation.

The major restriction of this study is that NIH/3T3 is a mouse fibroblast cell model, and the genetic background may not be correlated with epithelia, although NIH/3T3 has been recognized as a cell line for tumorigenesis studies in vitro and in vivo^17,55,56^. We could not observe EGFR expression in NIH/3T3 cells, which is similar to previous studies showing that NIH/3T3 cells lack EGFR^57,58^. Therefore, we tried to use a normal colon cell line, CCD-841CoN, as a model and found that acrolein indeed induced activation of the RAS/MAPK pathway, which was similar to NIH/3T3 (Fig. 3E). In addition, acrolein also induced cell proliferation, colony formation activity and cell migration capacity in CCD-841CoN cells (Fig. 4A-C). Activation of the RAS/MAPK signaling pathway was also observed in the CCD-841CoN Acr-clone (Fig. 4D). Furthermore, we found that acrolein increased the phosphorylation of EGFR, indicating that activation of EGFR results in the downstream RAS/MAPK pathway in CCD-841CoN (Figs. 3E, 4D).

Taken together, we found that acrolein induced oncogenic transformation using NIH/3T3 cells in a xenograft mouse model through upregulation of the RAS/MAPK pathway. In addition, higher acrolein-induced DNA damage (Acr-dG adducts) was observed in tumor tissues than in adjacent normal epithelial cells in CRC patients. Interestingly, increased Acr-dG levels were associated with better prognosis in CRC patients. To our knowledge, this is the first study to show that acrolein is important in oncogenic transformation through activation of the RAS/MAPK signaling pathway, contributing to colon carcinogenesis. Thus, this study provides insight for the early detection and prevention of colon cancer in the future.

## Materials and methods

### Cell culture and acrolein treatment

A mouse fibroblast cell line (NIH/3T3) and human normal colorectal cell CCD 841 CoN (ATCC CRL-1790) were purchased from ATCC and maintained in Dulbecco’s modified Eagle’s medium (DMEM) supplemented with 10% BCS and 15% FBS, respectively. Acrolein stock solution (Sigma-Aldrich) was prepared freshly before use. Cells at 70% confluency were treated with different concentrations of acrolein (0–10 μM) in complete culture medium for 1 months at 37 °C in the dark, and acrolein-containing medium was changed every two days.

### Cell proliferation assay

Cell proliferation was determined using modified 3-(4,5- dimethylthiazol-2-yl)-2,5-diphenyl tetrazolium (MTT; Sigma, St. Louis, MO) assay^59^. Briefly, cells (1000/well) were seeded in 96-well plates overnight and measured every day for 7 days. The resulting formazan dissolved in DMSO was measured at 570 nm, and the results are presented as the percentage of the control values. All of these experiments were performed in triplicate and were repeated independently at least three times.

### Flow cytometry analysis of cell cycle phases

Cells were washed twice in ice-cold PBS and fixed in ice-cold 70% ethanol for 30 min or overnight at 4 °C. Cells were then washed in PBS and digested with DNase-free RNase A (50 U/mL) at 37 °C for 30 min. Before flow cytometry analysis, cells were resuspended in 500 μL of propidium iodide (PI, 10 μg/mL; Sigma) for DNA staining. PI staining was used to measure the cell cycle status using a Becton–Dickinson FACScan instrument and Cell Quest software.

### Soft agar colony formation assay

A soft agar colony formation assay was performed as described previously^60^. Briefly, a 3-mL aliquot of 1.2% agar in culture medium was plated in 60-mm dishes. Then, 1,000 transformed malignant or untransformed cells were mixed with 3 mL of 0.35% agar in medium and plated on solidified bottom agar. When the top agar solidified, the dishes were transferred to an incubator and cultured for 30 days. Two or three drops of the medium were added to each dish three times a week. After culturing for 30 days, the visible cell colonies were photographed and counted.

### Tumor sphere culture assay

Acrolein-transformed NIH/3T3 clones were trypsinized and resuspended at 1000 cells/Ultra-Low Attachment 96-well Plate (Corning) in culture medium containing 2 mM l-glutamine, N2 supplement, B27 supplement, 20 ng/mL hrEGF (Sigma), and 20 ng/mL hrbFGF (Sigma) for two weeks. Fresh growth factors were added to the cells twice a week. Cumulative total numbers of cells from the spheroid cultures were calculated.

### Cell migration assay

The cell migration assay was performed in vitro utilizing modified Boyden chambers with a Transwell apparatus (polycarbonate membranes with 8-mm pores, Corning)^61^. Parental NIH/3T3 or NIH/3T3 Acr clones (5 × 10^4^ in 500 μL of growth medium/well, 6-well plates) were added to the upper chamber, and the lower chamber contained 750 μL of growth medium supplemented with 10% FCS. Cells on the upper membrane surface were wiped with a cotton swab after 24 h of incubation at 37 °C in a 5% CO_2_ incubator. Membranes were then fixed and stained with crystal violet, and cells that migrated to the lower membrane surface were counted in nine random fields using a microscope at 200 × magnification. These experiments were performed in triplicate and were repeated at least three times.

### Immunoblotting analysis

Cells were washed twice with ice-cold PBS and lysed on ice for 20 min in radioimmunoprecipitation assay (RIPA) lysis buffer (20 mM Tris HCl, 150 mM NaCl, 1% (v/v) NP-40, 1% (w/v) sodium deoxycholate, 1 mM ethylenediaminetetraacetate (EDTA), 0.1% (w/v) sodium dodecyl sulfate polyacrylamide (SDS) plus protease and phosphatase inhibitors). Lysates were then centrifuged at 13,200 rpm for 10 min, and the protein concentrations of supernatant were determined by a BCA Protein Assay Kit. Protein samples (30 μg) were run on 8–100% SDS–polyacrylamide gel electrophoresis and then transferred into polyvinylidene difluoride (Bio-Rad, U.S.A.) at 90 V for 120 min. Proteins were transferred onto nitrocellulose membranes (Bio-Rad). Blots were probed with primary antibodies overnight at 4 °C. Primary antibodies included: P-EGFR (Tyr1148, 1:1000, Cell Signaling #4404); EGFR (1:1000, Cell Signaling #2232); RAS (1:1000, Cell Signaling #3965); p-AKT (1:1000, Cell Signaling #4058); AKT (1:1000, Cell Signaling #4685); P-p44/42 MAPK (Erk1/2) (Thr202/Tyr204) (1:1000, Cell Signaling #9101); p44/42 MAPK (Erk1/2) (Thr202/Tyr204) antibody #9102; Cyclin D1 (1:1000, Cell Signaling #2978); c-myc (1:500, Santa Cruz, sc-42) and anti-acrolein antibody [10A10] (1:1000, Abcam, ab240918)) for acrolein-protein conjugates (Acr-PC). After primary antibody incubation, the membrane was washed and incubated with horseradish peroxidase-conjugated secondary IgG (1:3000; Millipore) for 1 h at room temperature. Immunoreactive bands were detected using Amersham Enhanced Chemiluminescence (Amersham Pharmacia Biotech, Piscataway, NJ, U.S.A.). The bound primary and secondary antibodies were stripped by incubating the membrane in stripping buffer (100 mM 2-mercaptoethanol, 2% SDS) for 30 min at room temperature. The membrane was then reprobed with GAPDH (1:1000, Cell Signaling, #5174).

### Slot blot assay for Acr-dG detection

Analysis of Acr-dG adducts in DNA samples was based on previously described methods^12,62^. Briefly, buccal DNA (0.25 μg) was loaded onto PVDF membranes using a Bio-Dot SF microfiltration apparatus (Bio-Rad, Hercules, CA). WesternDot 625 Western blotting kits (Invitrogen) were used for Western blot analysis according to the manufacturer’s instructions. The membrane was probed overnight at 4 °C with anti-Acr-dG mouse monoclonal antibodies^63^. Acr-dG adducts were detected using a UVP BioDoc-It imaging system, and band density was quantified with UVP imaging software. Relative Acr-dG levels were calculated by the fluorescence intensity of Acr-dG stained with an anti-Acr-dG antibody normalized to the amount of loaded DNA stained with methylene blue.

### Xenograft mouse model

Fifteen 6-week-old male Balb/c nude mice weighing 25–30 g were used. All animal experiments were approved by the Institutional Animal Care and Use Committee of National Yang-Ming University, and the study was carried out in compliance with the ARRIVE guidelines (IACUC#1070208rr). Tumors were induced by injecting acrolein-transformed NIH/3T3 cells (5 × 10^6^ in 100 μl of PBS per animal) subcutaneously into the right axillary fossa of mice as described previously with slight modification^61^. To generate the tumor growth curve, measurement of tumors was performed twice a week with a digital caliper, and volumes were calculated by (length x width^2^)/2. Body weight was also evaluated twice weekly. Tumor samples were collected after sacrifice. Each sample was cut in half; one half was saved in 10% formaldehyde, and one half was stored at − 80 °C until further use.

### RNA isolation and cDNA microarray analysis

Total RNA was isolated from TRIzol Reagent cells (Thermo Fisher Scientific) according to the manufacturer’s instructions. RNA samples were quantified using an ND-1000 spectrophotometer (NanoDrop Technologies, Wilmington, USA) The quality was determined using an Agilent 2100 Bioanalyzer with a Nanochip (Agilent, Santa Clara, CA) following the manufacturer's instructions. Microarray hybridizations were performed using total RNA prepared from NIH/3T3 and NIH/3T3 Acr-clone#4 cells as described previously^48^. GeneChip Mouse Genome 430 2.0 Affymetrix oligonucleotide gene chips (Affymetrix) were analyzed at the Microarray & Gene Expression Analysis Core Facility (VYM Genome Research Center, National Yang-Ming University) according to the Affymetrix protocols. Microarray datasets were analyzed using Ingenuity Pathway Analysis (IPA version 62089861) (QIAGEN), and bioinformatic analysis of the differentially expressed genes (DEGs, fold change ≥ 2) was performed. The IPA identified biological functions that were most significant to the data set. DEGs that were associated with biological functions in the Ingenuity Knowledge Base (Ingenuity Systems) were used for the analysis. Fisher’s exact test was used to calculate a p value that determined the probability that each biological function assigned to that network or to the data set was due to chance alone.

### Collection of tissue microarray of CRC patients

A total of 236 patients diagnosed with CRC at Taipei Veterans General Hospital were enrolled. Disease stage was assessed based on the American Joint Committee on Cancer staging system, 6th edition. Clinicopathological staging and clinical course were determined by searching a computer database containing detailed information. The medical residual samples of the patients were acquired from the residual sample bank of Taipei Veterans General Hospital, and this study was approved by the Institutional Review Board of Taipei Veterans General Hospital (VGHIRB, IRB#2020-01-010BC). VGHIRB waived the requirement for the use of informed consent. Patients were classified based on their primary tumor locations, including the right-sided colon (tumors originating in the cecum, ascending colon, hepatic flexure, and transverse colon), left-sided colorectum (tumors originating in the splenic flexure, descending colon, sigmoid colon, rectosigmoid junction, and rectum. Low-grade cancers have cancer cells that are well differentiated or moderately differentiated. High-grade cancers have cancer cells that are poorly differentiated or undifferentiated.

### Immunohistochemistry (IHC) analysis of the Acr-dG adduct and c-myc

For the tissue microarray (TMA), hematoxylin and eosin-stained sections from each paraffin-embedded, formalin-fixed block were used to define diagnostic areas, and a representative 0.6 mm core was obtained from each case and inserted in a grid pattern into a recipient paraffin block^49,50^. IHC analysis was carried out as previously described with slight modification^64^. Briefly, sections (4 µm) were then deparaffinized in xylene and rehydrated in a descending ethanol series. To enhance immunoreactivity, sections were incubated in Tris–EDTA, pH 6.0, and boiled for 12 min. Endogenous peroxidase activity was eliminated by incubation in hydrogen peroxide. Incubation with primary antibodies for Acr-dG antibody (generated in house) and c-myc (Santa Cruz, sc-40) was performed overnight at 4 °C in 1% BSA in phosphate-buffered saline (PBS). Bound antibodies were visualized with DAB (diaminobenzidine) used as a chromogen, and omission of the primary antibody served as a negative control. Positive controls (normal liver) were stained in parallel with each set of TMAs studied. Assessment of Acr-dG and c-myc immunoexpression was performed by light microscopy at × 400 magnification by a pathologist.

### Statistical analyses

Descriptive statistics are presented as the mean ± standard deviation or as the number (percentage). Student’s t tests were used to determine statistical significance, and two-tailed P-values are shown. A minimum of three independent replicate experiments was performed to justify the use of statistical tests. Survival was analyzed using Kaplan–Meier survival analysis, and the log rank test was used for comparison between the two groups. Multivariate analysis was performed using chi-square analysis or Fisher's exact test. Statistical significance was defined as a *p* < 0.05. All analyses were performed with the IBM SPSS Statistics software package, version 23.0 or R software (R version 4.1.0).

### Ethics approval

Our study protocol was approved by the Institutional Review Board of Taipei Veterans General Hospital (IRB#2020-01-010BC) and the study was carried out in accordance with the Declaration of Helsinki principles. All animal experiments were approved by the Institutional Animal Care and Use Committee of National Yang-Ming University and was carried out in compliance with the ARRIVE guidelines (IACUC#1070208rr).

## Supplementary Information

Supplementary Information.
