# Author Correction: Acrolein contributes to human colorectal tumorigenesis through the activation of RAS-MAPK pathway

**DOI:** 10.1038/s41598-021-95257-3

**Published:** 2021-08-02

**Authors:** Hong-Chieh Tsai, Han-Hsing Tsou, Chun-Chi Lin, Shao-Chen Chen, Hsiao-Wei Cheng, Tsung-Yun Liu, Wei-Shone Chen, Jeng-Kai Jiang, Shung-Haur Yang, Shih-Ching Chang, Hao-Wei Teng, Hsiang-Tsui Wang

**Affiliations:** 1 Department of Neurosurgery, Linkou Chang Gung Memorial Hospital, Taoyuan, 333 Taiwan; 2 School of Traditional Chinese Medicine, Chang Gung University, Taoyuan, 333 Taiwan; 3 Institute of Food Safety and Health Risk Assessment, National Yang-Ming University, Taipei,, Taiwan; 4 Institute of Food Safety and Health Risk Assessment, National Yang Ming Chiao Tung University, Taipei, Taiwan; 5 Kim Forest Enterprise Co., Ltd., Taipei, Taiwan; 6 School of Medicine, National Yang-Ming University, Taipei, Taiwan; 7 School of Medicine, National Yang Ming Chiao Tung University, Taipei, Taiwan; 8 Division of Colon and Rectum Surgery, Department of Surgery, Taipei Veterans General Hospital, Taipei, Taiwan; 9 Institute of Pharmacology, College of Medicine, National Yang-Ming University, Taipei, Taiwan; 10 Institute of Pharmacology, College of Medicine, National Yang Ming Chiao Tung University, Taipei, Taiwan; 11 Department of Surgery, National Yang Ming Chiao Tung University Hospital, Yilan, Taiwan; 12 Division of Medical Oncology, Department of Oncology, Taipei Veterans General Hospital, Taipei, Taiwan

Correction to: *Scientific Reports* 10.1038/s41598-021-92035-z, published online 15 June 2021

The original version of this Article contained an error in the spelling of the author Hsiang-Tsui Wang which was incorrectly given as Hsiang-Tsai Wang. The original Article and accompanying Supplementary Information file have been corrected.

