# Supplementary material for: Acrolein contributes to human colorectal tumorigenesis through the activation of RAS-MAPK pathway
Source: Sci Rep. 2021 Jun 15;11:12590. doi: 10.1038/s41598-021-92035-z (PMC8206110; doi:10.1038/s41598-021-92035-z)
Supplement: Supplementary file 1 — Supplementary Information. [file 41598_2021_92035_MOESM1_ESM.docx]

**Acrolein contributes to human colorectal tumorigenesis through the activation of RAS-MAPK pathway**

**Hong-Chieh Tsai^1,2^, Han-Hsing Tsou^3^, Chun-Chi Lin^4,5^, Shao-Chen Chen^3^, Hsiao-Wei Cheng^1,6^, Tsung-Yun Liu^3^, Wei-Shone Chen^4,5^, Jeng-Kai Jiang^4,5^, Shung-Haur Yang^4,5,7^, Shih-Ching Chang^4,5^, Hao-Wei Teng^4,8,9^*, Hsiang-Tsui Wang^6^***

**Supplementary Tables**

**Table 1. Twelve genes associated with colorectal cancer metastasis signaling using IPA canonical pathway analysis.**

| **Symbol** | **Entrez Gene Name** | **Expr Fold Change^a^** | **Location** | **Type(s)** |
| --- | --- | --- | --- | --- |
| MMP14 | matrix metallopeptidase 14 | -2.27 | Extracellular Space | peptidase |
| MYC | MYC proto-oncogene, bHLH transcription factor | 2.21 | Nucleus | transcription regulator |
| PGF | placental growth factor | 2.45 | Extracellular Space | growth factor |
| PIK3CB | phosphatidylinositol-4,5-bisphosphate 3-kinase catalytic subunit beta | 2.14 | Cytoplasm | kinase |
| PIK3R1 | phosphoinositide-3-kinase regulatory subunit 1 | -4.61 | Cytoplasm | kinase |
| PTGER4 | prostaglandin E receptor 4 | 2.09 | Plasma Membrane | G-protein coupled receptor |
| PTGS2 | prostaglandin-endoperoxide synthase 2 | 6.83 | Cytoplasm | enzyme |
| RHOU | ras homolog family member U | -2.14 | Cytoplasm | enzyme |
| RND1 | Rho family GTPase 1 | 3.63 | Cytoplasm | enzyme |
| RRAS2 | RAS related 2 | 2.69 | Plasma Membrane | enzyme |
| TGFB2 | transforming growth factor beta 2 | -7.13 | Extracellular Space | growth factor |
| TLR2 | toll like receptor 2 | -2.92 | Plasma Membrane | transmembrane receptor |
| ^a^ Expression fold change: RNA expression fold change between NIH/3T3 Acr-clone#4 and parental cells. | | | | |

**Supplementary Table 2. Multivariable Cox regression models.**

|  | HR | 95% CI | P-value |
| --- | --- | --- | --- |
| Age | 1.143 | (0.775-1.685) | 0.499 |
| Location | 0.717 | (0.494-1.039) | 0.079 |
| Gender | 0.844 | (0.573-1.245) | 0.394 |
| acrolein | 0.698 | (0.483-1.008) | 0.055 |
| stage | 1.086 | (1.058-1.114) | <0.001 |
| Grade | 1.081 | (0.573-2.041) | 0.809 |
| LVSI | 1.653 | (1.110-2.462) | 0.013 |
| LVSI: lymph-vascular space invasion. | | | |

**Supplementary Table 3. The proportional hazards assumption test for a Cox regression model.**

|  | Chi-square test | df | P-value |
| --- | --- | --- | --- |
| Gender | 0.705 | 1 | 0.40 |
| Location | 0.362 | 1 | 0.55 |
| stage | 0.148 | 1 | 0.70 |
| age | 0.549 | 1 | 0.46 |
| Grade | 1.625 | 1 | 0.2 |
| LVSI | 0.153 | 1 | 0.70 |
| Acr-dG | 0.576 | 1 | 0.45 |
| GLOBAL | 3.898 | 7 | 0.79 |
| LVSI: lymph-vascular space invasion. | | | |

**Supplementary Table 4. A plot of scaled Schoenfeld residuals (y-axis) against (transformed) event time (x-axis) for a Cox proportional hazards model.**

| **Gender**   | **Location**   |
| --- | --- |
| **Stage**   | **Age**   |
| **Grade**   | **LVSI (lymph-vascular space invasion.)**   |
| **Acr-dG**  |  |

**Supplementary Figures**

**
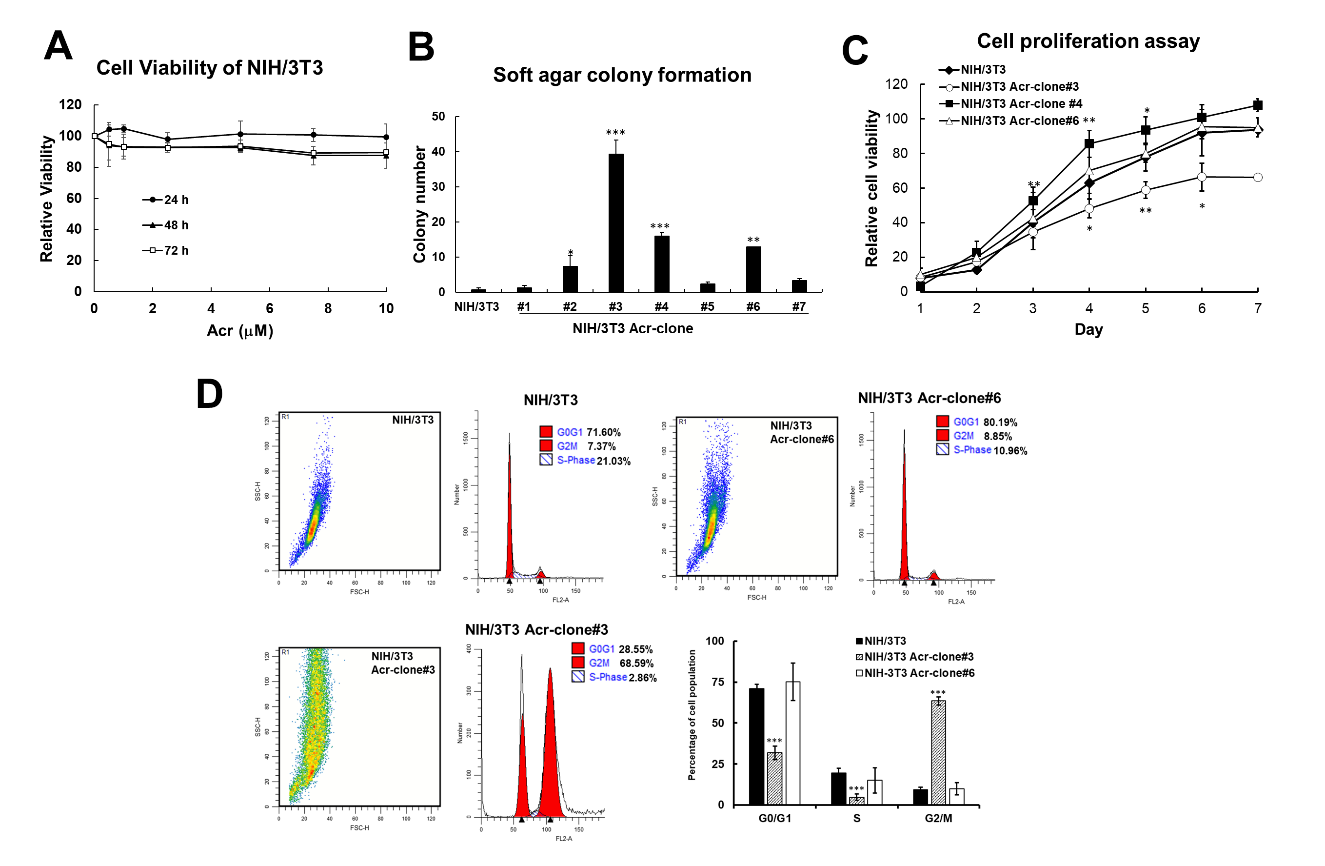
**

**Supplementary Figure 1. Selection of NIH/3T3 Acr-clones using low dose of acrolein treatment for 1 months.** NIH/3T3 cells were treated acrolein (Acr, 7.5 μM) for one month and named as NIH/3T3 Acr-clone#. (A) Cell viability of NIH/3T3 under low dose of acrolein (0-10 μM) treatment for 1-3 days was analyzed using MTT assays. (B) Anchorage independent cell growth of NIH/3T3 Acr-clone #1-7 was analyzed using soft agar assay. (C) Cell proliferation of NIH/3T3 Acr-clone #3, #4 and #6 compared with parental cells was analyzed using MTT assays. (D) cell cycle progression of NIH/3T3 Acr-clone #3 and Acr-clone#6 was analyzed using cell cycle analysis with PI staining.

**
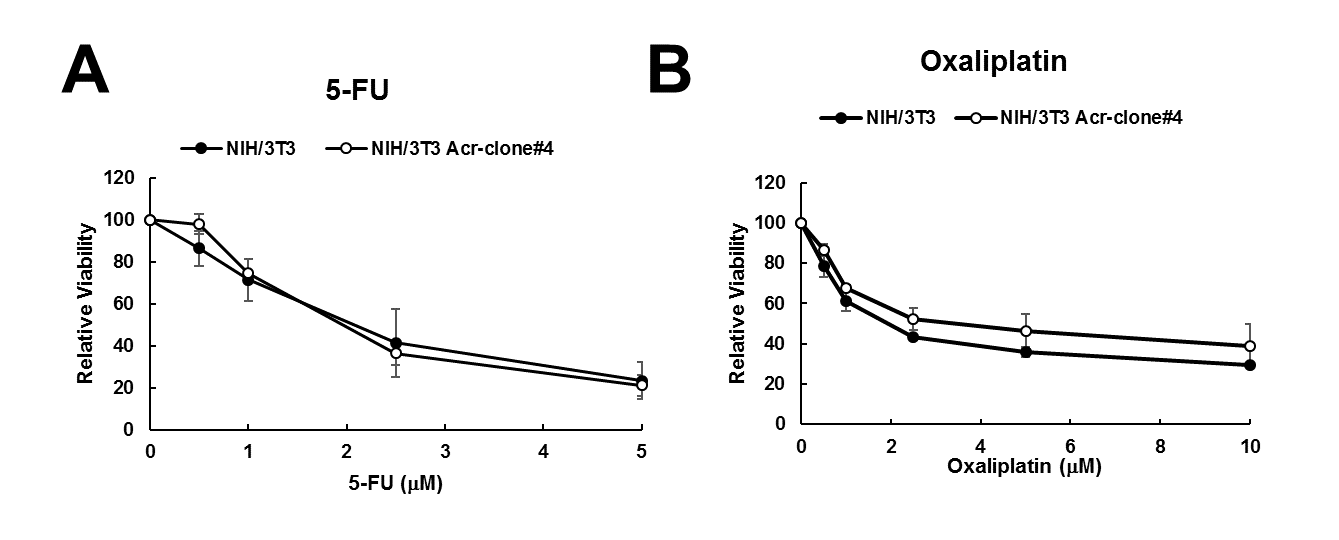
**

**Supplementary Figure 2. Cytotoxicity of 5-FU and Oxaliplatin in NIH/3T3 parental and NIH/3T3 Acr-clone#4.** Cells were treated with 5-FU (0-5 μM) and oxaliplatin (0-10 μM) for 24 h followed by MTT analysis as described in Materials and methods.


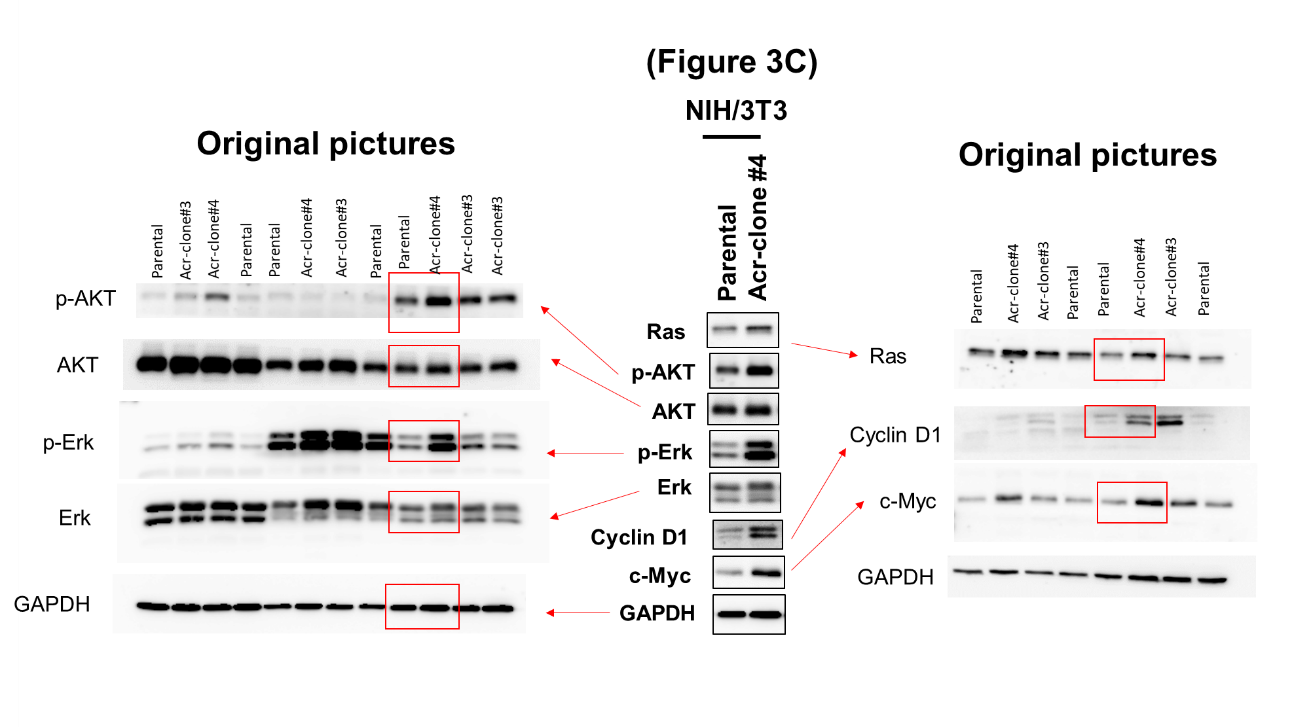


**Supplementary Figure 3A. Original Western blots of Figure 3C.** The grouping of blots was cropped from the same samples loaded in two gels with 9 blots.

**
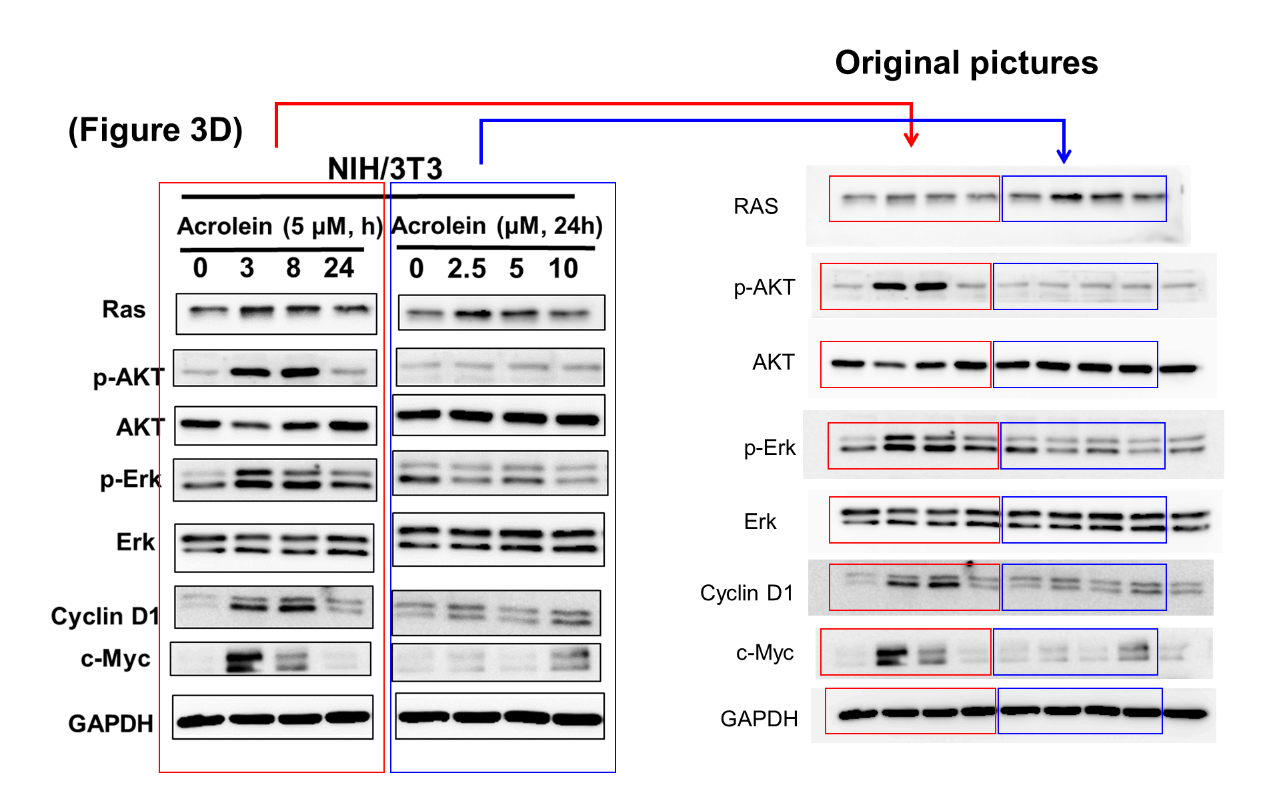
**

**Supplementary Figure 3B. Original Western blots of Figure 3D.** The grouping of blots was cropped from the same samples loaded in two gels with 8 blots.

**
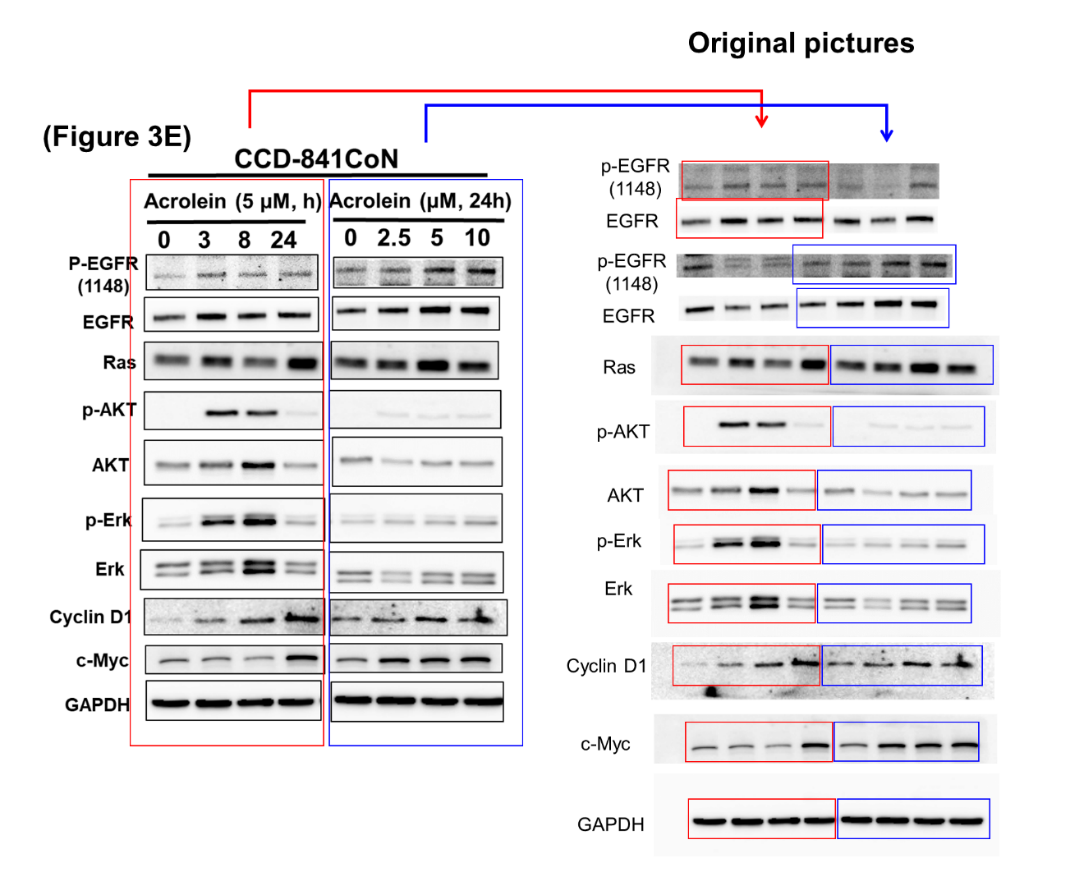
**

**Supplementary Figure 3C. Original Western blots of Figure 3E.** The grouping of blots was cropped from the same samples loaded in four gels with 12 blots.

**
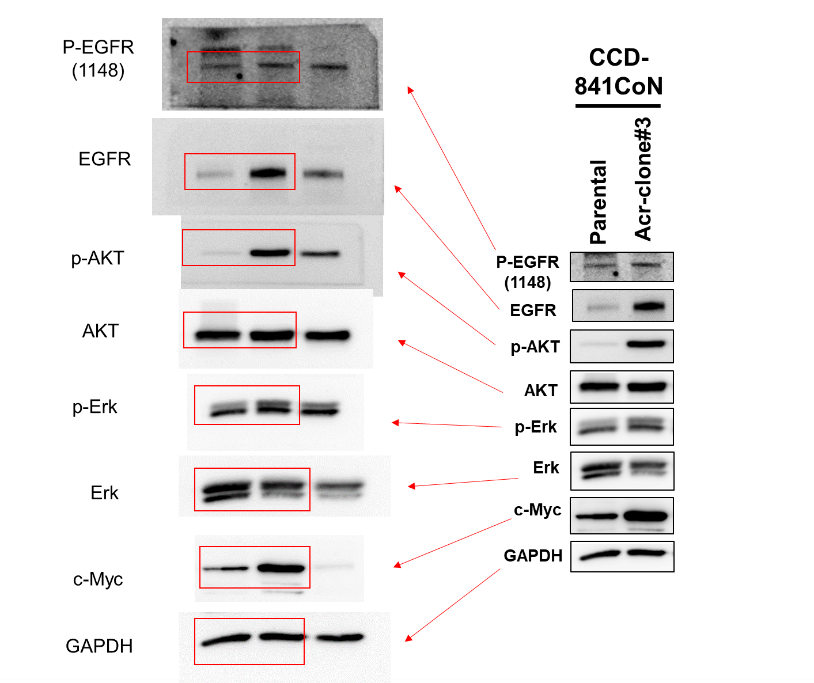
**

**Supplementary Figure 3D. Original Western blots of Figure 4E. The grouping of blots was cropped from the same samples loaded in two gels with 8 blots.**


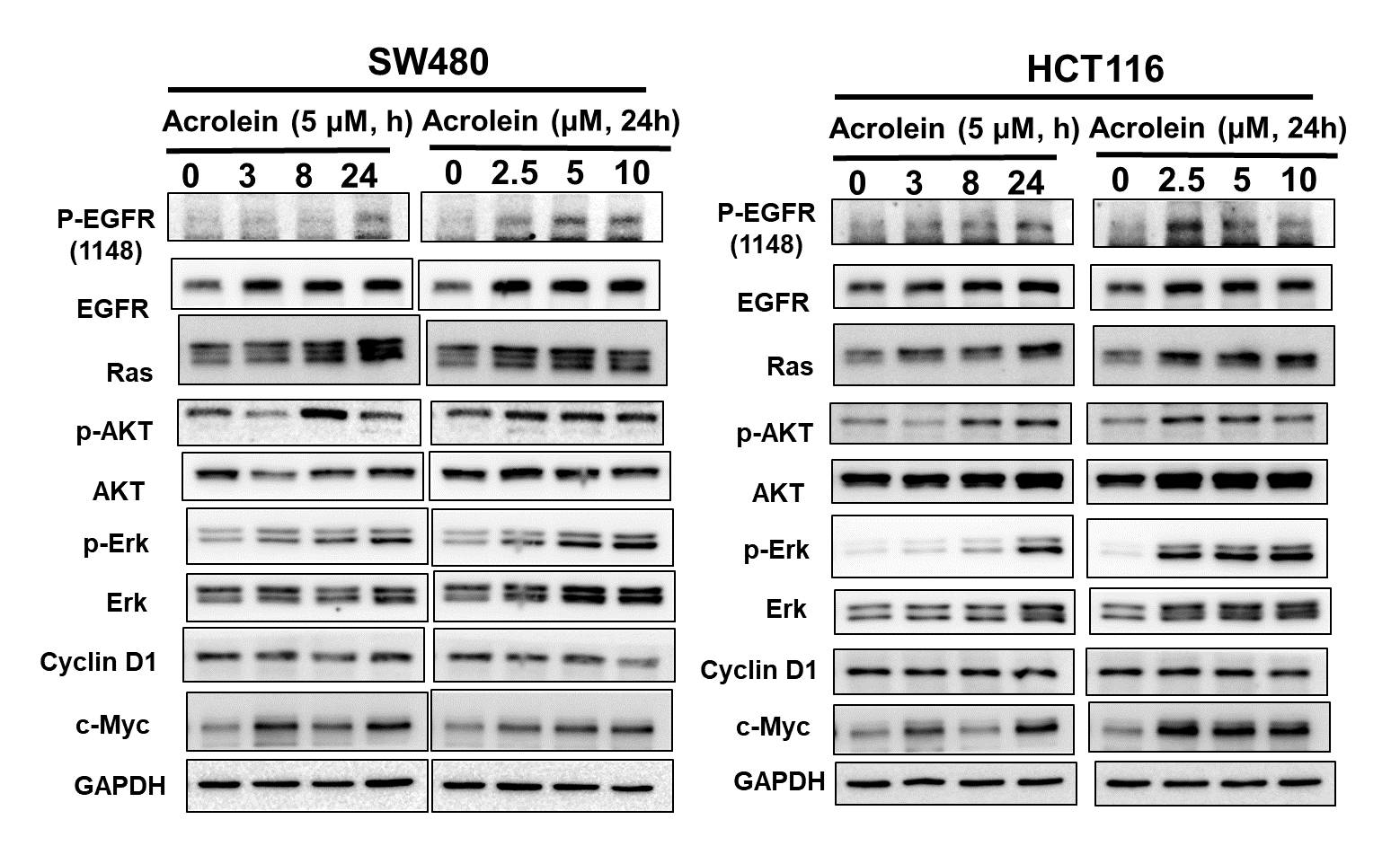


**Supplementary Figure 4. Acrolein activated RAS/MAPK pathway in human colorectal cancer cell line, SW480 and HCT116.** Dose and time effects of acrolein on RAS expression, AKT activation, ERK activation, cyclin D1 and c-myc expression in SW480 and HCT116 cells were analyzed using western blot analysis. For dose and time effect, cells were treated with different concentrations of acrolein (0-10 μM) for 24 h or acrolein (5 μM) for 3-24 h, respectively


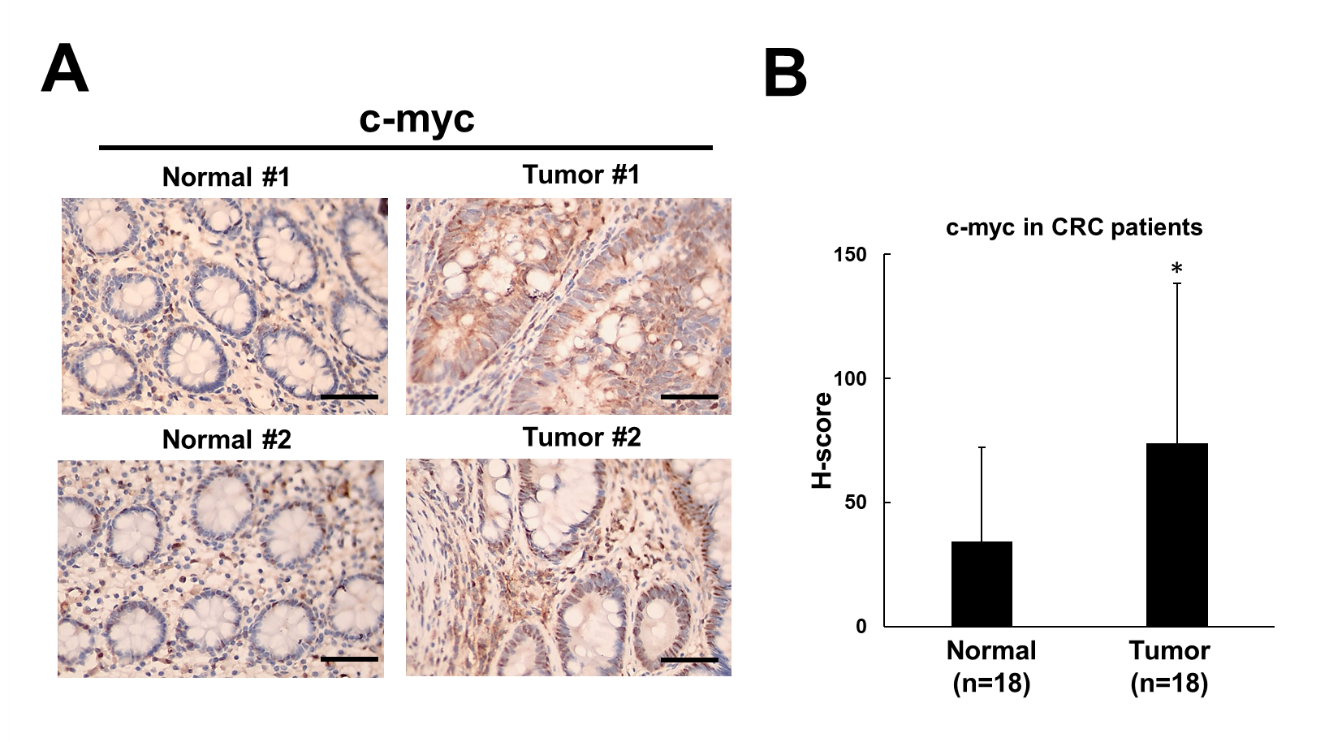


**Supplementary Figure 5. Immunohistochemical staining for c-myc in eighteen CRC patients.** (A) Representative image of c-myc in normal epithelial cells adjacent to the CRC tumor tissues. (B) quantification of c-myc in normal epithelial cells adjacent to the CRC tumor tissues (magnification, ×400). Scale bar: 50 μM. Student’s *t* tests were used to determine statistical significance, and two-tailed p-values are shown. *p<0.05 compared between tumor tissues and normal tissues.


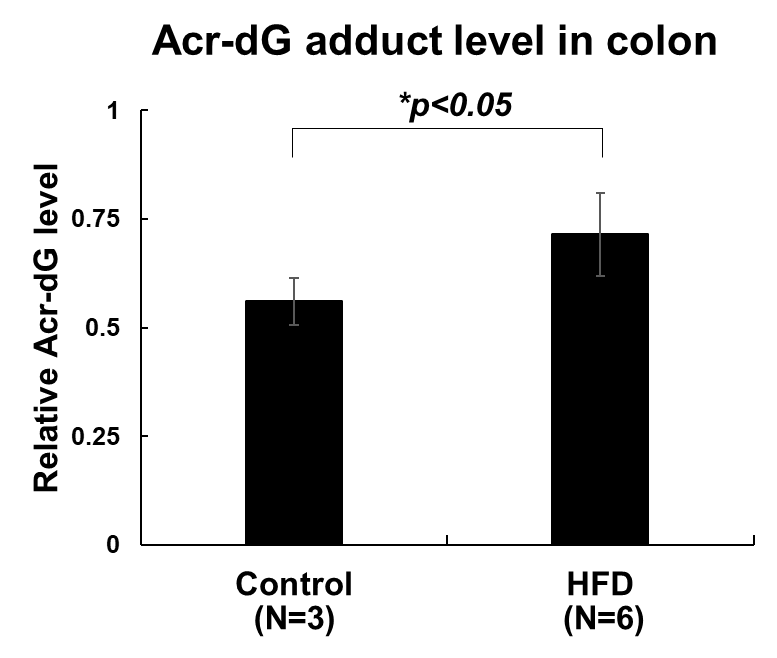


**Supplementary Figure 6. Slot blot analysis for Acr-dG adducts in colon tissues of mice with high fat diet (HFD).** Relative Acr-DNA adduct levels in colon DNA of mice fed with HFD for 24 weeks (n=6) and normal diet (n=3) using slot blot analysis as described in Materials and methods. 6-week-old male Balb/c nude mice, weighing 25-30 g, were fed with high fat diet for 24 weeks and colon tissues were collected after sacrifice. All animal experiments were approved by the Institutional Animal Care and Use Committee of National Yang-Ming University.


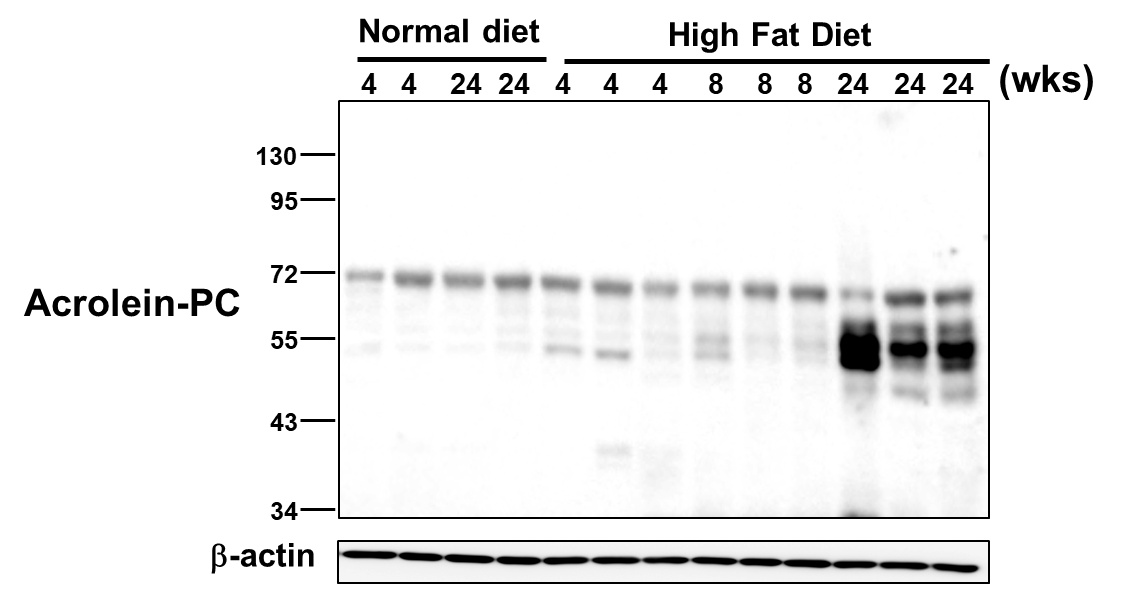


**Supplementary Figure 7. Expression of acrolein-protein conjugates in colon tissues of mice fed with high fat diet (HFD).** Western blot analysis of acrolein-protein conjugates (Acr-PC) in mixed colon tissues of mice fed with HFD for 4-24 weeks (n=3) compared to mice fed with normal diet (n=2). Animal studies were performed as described in Supplementary Figure 4.

**
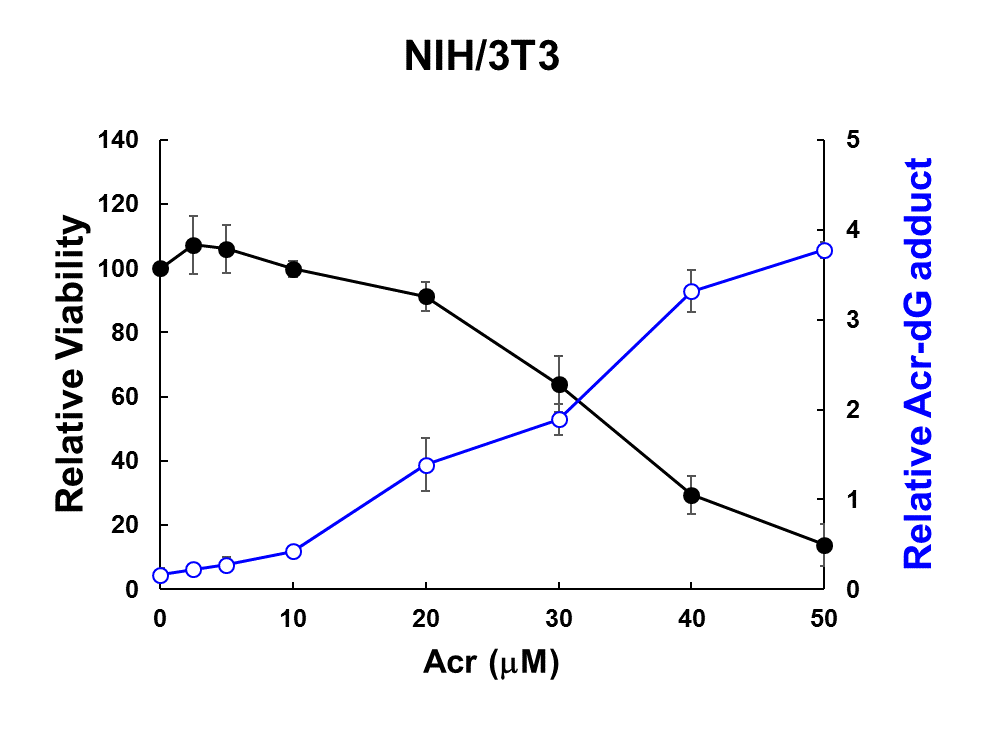
**

**Supplementary Figure 8. Cytotoxicity and genotoxicity of acrolein in NIH/3T3 cells.** NIH/3T3 cells were treated with acrolein (0-150 μM) for 24 h. Cytotoxicity was analyzed using MTT analysis. Genotoxicity of acrolein was shown as relative Acr-dG adduct levels using slot blot analysis. Inverse correlation between cell viability and relative Acr-dG adduct levels was observed.

**
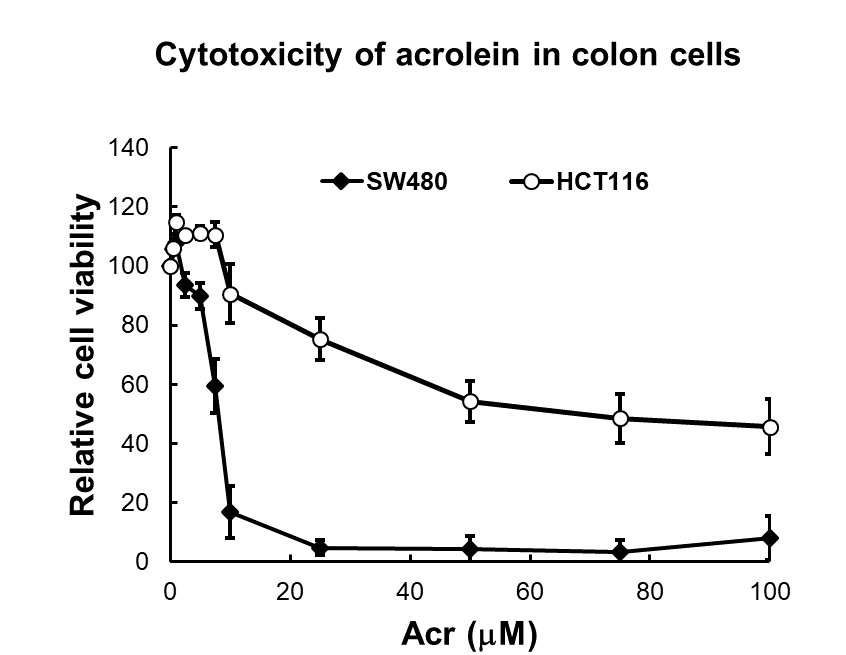
**

**Supplementary Figure 9. Cytotoxicity of acrolein in human colon cancer cell lines, SW480 and HCT116.** Cells were treated with acrolein (0-100 μM) for 24 h followed by MTT analysis as described in Materials and methods.
